# Study of Estrogen Receptor-Mediated PGx-eQTLs Identifies Genetic Determinants of Breast Cancer Endocrine Therapy Response

**DOI:** 10.3390/ijms27188217

**Published:** 2026-09-15

**Authors:** Martin Meng, Arnab Ghosh, Huanyao Gao, John August, Shreya Indulkar, Meijie Wang, Xue Wang, Zhuyao Wang, Asadoor Amirkhani Namagerdi, Richard M. Weinshilboum, James N. Ingle, Liewei Wang

**Affiliations:** 1 Department of Molecular Pharmacology and Experimental Therapeutics, Mayo Clinic, Rochester, MN 55905, USA; meng.martin@mayo.edu (M.M.); arnab.ghosh@flinders.edu.au (A.G.); huanyao.gao@yale.edu (H.G.); augiejohn2024@gmail.com (J.A.); indulkar.shreya@mayo.edu (S.I.); wang.meijie@mayo.edu (M.W.); wang.xue2@mayo.edu (X.W.); wang.zhuyao@mayo.edu (Z.W.); amirkhaninamagerdi.asadoor@mayo.edu (A.A.N.); weinshilboum.richard@mayo.edu (R.M.W.); 2 Flinders Health and Medical Research Institute, Flinders Centre for Innovation in Cancer, Flinders University, Adelaide, SA 5042, Australia; 3 Yale School of Medicine, Yale University, New Haven, CT 06510, USA; 4 Department of Medicine-Hematology/Oncology, University of California San Francisco, San Francisco, CA 94143, USA; 5 Department of Oncology, Mayo Clinic, Rochester, MN 55905, USA; ingle.james@mayo.edu

**Keywords:** GWAS, pharmacogenomics, estrogen receptor, tamoxifen, PGx-eQTL, breast cancer, endocrine therapy

## Abstract

Endocrine therapy remains the cornerstone of treatment for estrogen receptor alpha (ERα)-positive breast cancer, yet the relationship between individual genetic background and molecular response to ERα-targeted therapy remains incompletely understood. Using a well-characterized panel of lymphoblastoid cell lines (LCLs), we performed genome-wide pharmacogenomic expression quantitative trait locus (PGx-eQTL) analysis to identify estradiol (E2)- and tamoxifen (TAM)-induced SNP-gene pairs. PGx-eQTL signals were integrated with previously published breast cancer genome-wide association study datasets to examine their association with clinically relevant breast cancer phenotypes. We identified two ER-mediated PGx-eQTL SNP-gene pairs associated with breast cancer prognosis post-treatment with E2 or TAM. Notable loci included E2-regulated *MRPL15* and TAM-regulated *SYCP3*, which have effects in a genotype-dependent manner, with genotype-dependent survival outcomes, from worse to better relapse-free survival. Similar endocrine-therapy effects on patient survival and breast cancer risk were observed in *SIK2*, post TAM-treatment, and in *LSM4* with E2-treatment. Moreover, qRT-PCR validation in an independent LCL panel confirmed the genotype-specificity of these signals. Overall, we identified ER-mediated PGx-eQTL SNP-gene pairs which represent potential pharmacogenomic tools for identifying patients likely to benefit from ERα-targeted endocrine therapy, offering a foundation for more genotype-informed individualized treatment decisions in breast cancer.

## 1. Introduction

Breast cancer is the second leading cause of death among women and the most prevalent cancer globally. In 2025, breast cancer represented 15.50% of all new cancer cases in the United States, with an estimated 316,950 new cases [1]. In the United States, it is the most frequently diagnosed cancer and the leading cause of death in women under 50. The majority of breast cancers in these patients are estrogen receptor α-positive (ERα+). Thus, endocrine therapy, including ER antagonists such as tamoxifen (TAM), a selective ER modulator (SERM), selective ER degraders (SERDs), as well as estrogen biosynthesis inhibitors such as aromatase inhibitors (AIs), has been widely used clinically in adjuvant, neo-adjuvant, and preventative settings. Despite their established efficacy, there is considerable interpatient variability in therapeutic response, adverse effects, and long-term outcomes that remains incompletely understood on a genetic and biological basis. To address this, genome-wide association studies (GWAS) have been important tools that have revealed single-nucleotide polymorphisms (SNPs) linked to several endocrine therapy treatment response phenotypes, such as disease-free survival in patients treated with aromatase inhibitors (AIs), breast cancer prevention efficacy in patients treated with tamoxifen (TAM), musculoskeletal adverse effects and fragility bone fractures in women on AIs, and suppression of estrone (E1) and estradiol (E2) plasma levels in women receiving AI anastrozole as adjuvant therapy. Together, these studies identified SNPs that act as expression quantitative trait loci (eQTLs) specifically in response to ERα ligands, helping to explain individual variability in endocrine therapy response [2,3,4,5,6].

An important consideration in the context of breast cancer endocrine therapy is the emergence of ER mutations, particularly SNPs that alter the function of the *ESR1* gene, which plays a crucial role in disease progression and the development of treatment resistance in hormone receptor-positive breast cancers. The other major ER, *ESR2***,** has mutations that are far less frequent and have been less consistently linked to endocrine resistance. Somatic *ESR1* mutations are most commonly observed in metastatic breast cancer following prolonged AI treatment, where they represent a key mechanism of acquired resistance. In this setting, liquid biopsy has emerged as a minimally invasive and clinically valuable tool for detecting *ESR1* mutations, particularly in hormone receptor-positive metastatic breast cancer after AI therapy, enabling the timely identification of hormone resistance and guiding necessary adjustments to endocrine treatment strategies [7,8,9]. The mechanisms through which *ESR1* alterations drive endocrine resistance are multifaceted; the primary mechanism involves constitutive, ligand-independent activation of the ER. Beyond this, other contributing factors include downstream pathway activation alternative to ER and disruption of the normal ER feedback loop, as well as enhanced sensitivity to non-estrogenic ligands. Interestingly, some studies showed that mutations in *ESR1* can be associated with broader changes in the tumor microenvironment by inducing angiogenesis, immune evasion mechanisms and altered cell adhesion to collectively facilitate metastatic proliferation of cancer cells [7,8].

Among the most widely used and clinically significant endocrine agents is TAM, the first SERM to be developed and the most widely prescribed to date. SERMs function as competitive inhibitors of estrogen towards ER binding and selectively modulate ER activity in a tissue-dependent manner, thus acting as ER antagonists in breast tissue while exerting agonistic estrogen-like effects in other tissues such as bone, where they promote bone mineralization and reduce osteoclastic activity. In breast cancer specifically, TAM competes with estrogen for binding to ERα (*ESR1*), partially blocking activation of the AF-1 domain of the ERα isoform, thereby inhibiting ER signaling and suppressing tumor cell growth [10,11,12,13]. At the molecular level, E2-bound ERα has been shown to induce enhancer-promoter looping and higher-order chromatin recompartmentalization to coordinate transcriptional responses, while altered ER chromatin organization has been closely linked to endocrine resistance. In contrast, TAM binding to ERα induces a distinct receptor conformation, blocking coactivator recruitment and engaging a fundamentally different transcriptional program, providing a strong biological rationale for studying both E2- and TAM-specific PGx-eQTL signals in parallel [14].

ER signals through ERα, which is a ligand-activated nuclear receptor; upon ligand binding, ERα undergoes nuclear localization and binds to specific DNA sequence motifs, ultimately regulating the transcription of target genes. Thus, genetic variants such as SNPs that alter DNA sequence can therefore modulate ER transcriptional activity in a genotype-dependent manner, which eventually alters the ER signaling pathway and its effect on cells. Critically, because ER transcriptional activity is heavily ligand-dependent, the influence of these SNPs on gene expression remains largely silent in the absence of ligand and, in this scenario, these ligand-dependent SNPs are referred to as pharmacogenomic eQTLs (PGx-eQTLs), in contrast to baseline eQTLs that are independent of pharmacological treatment. Previous studies identified SNP-gene pairs of PGx-eQTL signals implicated in drug response in breast cancer and psychiatric disorders, where the androgen receptor (AR) and the glucocorticoid receptor (GR) were identified as transcription factors. Historically, the gold standard for PGx-eQTL identification used lymphoblastoid cell lines (LCLs), because LCLs maintain a high degree of single-nucleotide polymorphism (SNP) accuracy, typically achieving a rate between 98.8% and 99.9% when compared directly to the donor’s original whole blood DNA. Thus, knowing exact genetic information about an individual’s DNA, including SNPs, made LCLs a more clinically relevant model than a well-established breast cancer cell line to study pharmacogenomic eQTLs [3,15,16,17,18,19].

In this manuscript, we have applied the same well-established strategy to interrogate ERα and identify PGx-eQTL SNP-gene pairs induced by either the ERα agonist E2 or the SERM TAM. These ER-mediated PGx-eQTL signals were then integrated with our previously published breast cancer GWAS data, with the goal of identifying silent non-coding genetic risk variants relevant to breast cancer prognosis, risk, and endocrine therapy response. The identification of such SNP-gene pairs may ultimately provide a foundation for more individualized approaches to endocrine therapy in breast cancer.

## 2. Results

### 2.1. Landscape of PGx-eQTLs Induced by ER Modulators

**Figure 1:**
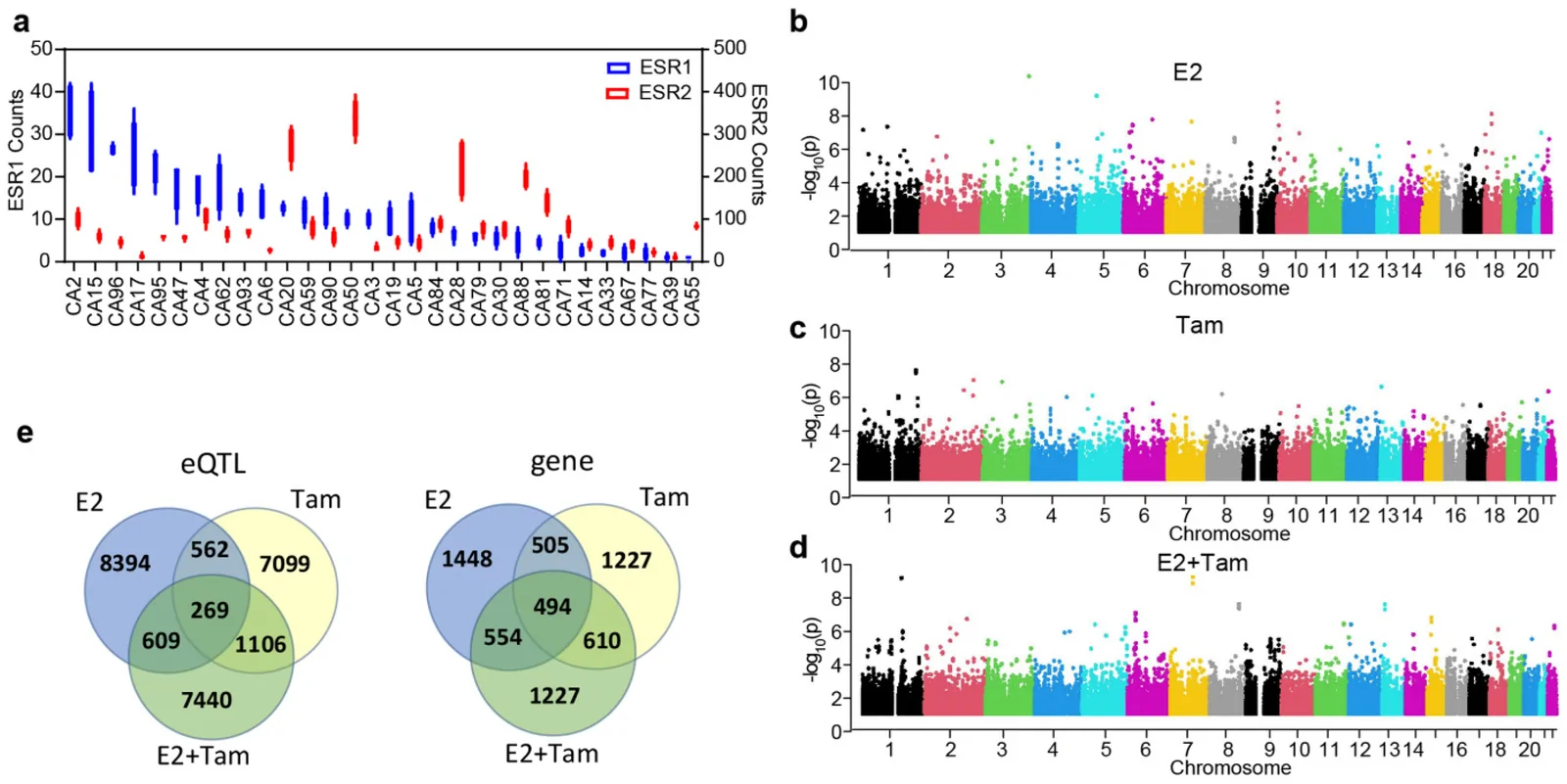
Identifying ER PGx-eQTL hits. (**a**) ESR1 (left y-axis) and ESR2 expression (right y-axis) in 30 LCLs based on RNA-seq counts. Each box represents ER expression in a specific LCL, as labeled at the bottom. (**b**) Manhattan Plots representing estradiol-induced PGx-eQTL signals in LCLs. The X-axis depicts the chromosomal location, and the y-axis depicts −log10(*p*-values) from the PGx-eQTL results. (**c**) Manhattan Plots representing tamoxifen-induced PGx-eQTL signals in LCLs. The X-axis depicts the chromosomal location, and the y-axis depicts −log10(*p*-values) from the PGx-eQTL results. (**d**) Manhattan Plots representing estradiol + tamoxifen-induced PGx-eQTL signals in LCLs. The X-axis depicts the chromosomal location, and the y-axis depicts −log10(*p*-values) from the PGx-eQTL results. (**e**) Venn diagrams comparing eQTL signals (**left**) and genes (**right**) among E2, Tam, and E2 + Tam conditions.

The stimulatory or repressive effects of estrogens are mediated through ERα and ERβ, which are gene products of *ESR1* and *ESR2*, respectively. In addition to these classical nuclear receptors, TAM, being an estrogen modulator/antagonist, is also believed to act through the distinct membrane-bound G protein-coupled estrogen receptor, GPER1 (also known as GPR30), which is structurally and evolutionarily unrelated to *ESR1*/*ESR2* [20]. Thus, to confirm ER-responsiveness, we analyzed the expression of *ESR1* and *ESR2* quantified by RNA-seq across 30 LCLs, and there was significant upregulation of both receptors with variable expression between different LCLs (Figure 1a). Further, analysis of differential PGx-eQTL between the initial landscape of signals prior to further filtering between E2-induced signals, TAM-induced signals, and E2 + TAM signals as presented through Miami plots confirmed that tamoxifen reversed the transcriptional changes induced by E2 (Figure 1b–d). It is well known that E2 and TAM compete for ER binding and therefore antagonize one another in ER signaling, yet when applied individually, each can regulate distinct subsets of genes. We therefore used expression profiles from the E2 + TAM dual-treatment condition to define drug specificity of E2- or TAM-induced signals. A significance threshold of *p* ≤ 0.005 was applied for PGx-eQTL analysis, in part reflecting the constraints of sample size. Using this cutoff, only 10–15% of eQTL SNP-gene pairs (878 out of 9834 for E2 and 1375 out of 9036 for TAM), along with around 35% of the eQTL genes (1048 out of 3001 for E2 and 1104 out of 2836 for TAM), were excluded, as their effects were not reversed under the dual-treatment condition (Figure 1e).

**Figure 2:**
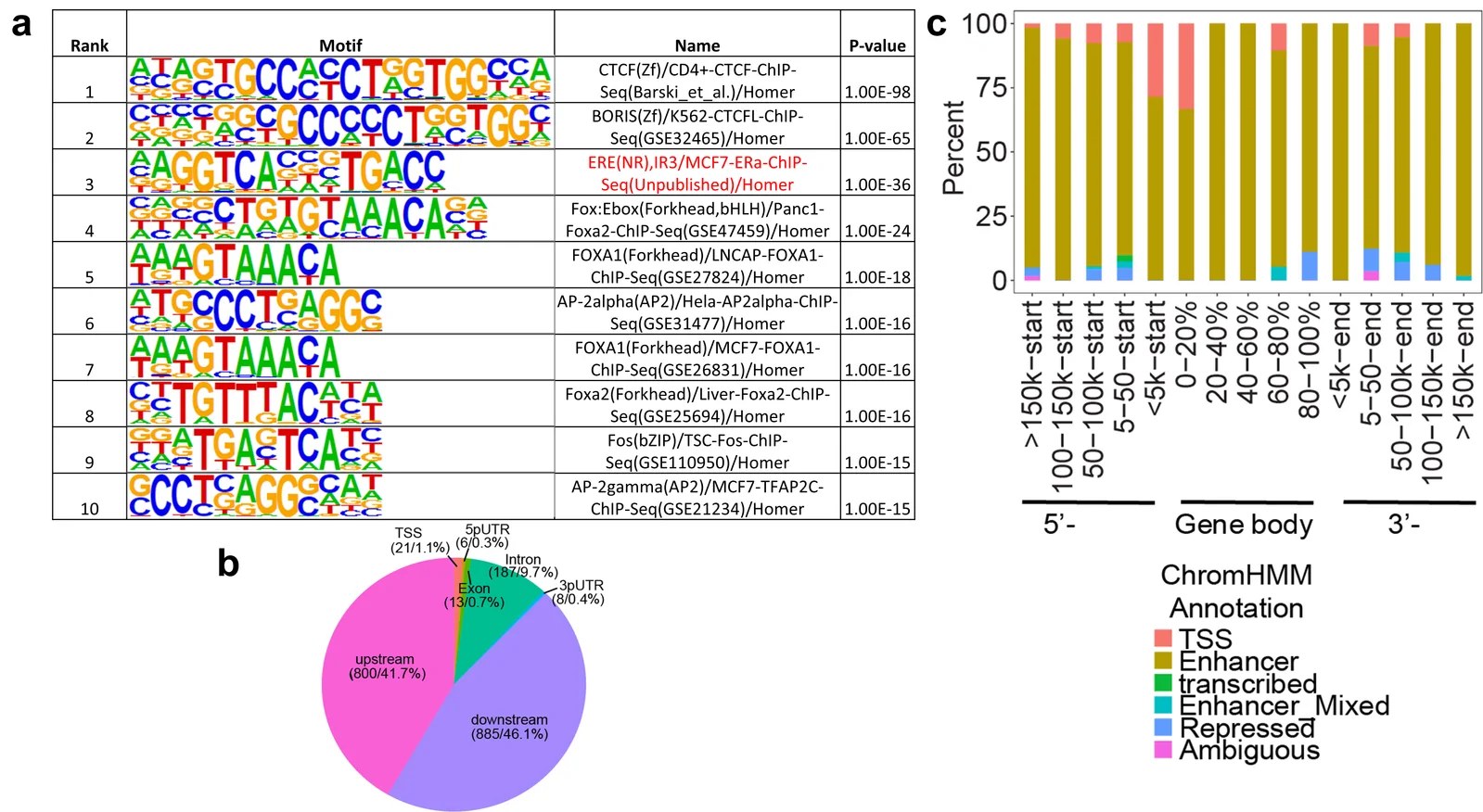
Analysis of cis-regulatory elements along with signals of ER PGx-eQTL. (**a**) Homer motif enrichment analysis performed on sequences flanking SNPs from E2/TAM-induced pharmacogenomic eQTL (PGx-eQTL) signal and also filtered against ER ChIP-seq binding sites. The classical ER binding motif is highlighted in red text. (**b**) Pie chart of regions occupied by SNP locations relative to the corresponding PGx-eQTL genes, filtered by ER ChIP-seq binding sites. (**c**) ChromHMM cis-regulatory elements (RE) analysis was performed at SNP sites using all E2/TAM-induced PGx-eQTL hits and filtered against ER ChIP-seq binding sites. The x-axis represents the relative distance of each SNP from its corresponding PGx-eQTL genes, while the color denotes cis-RE annotation assigned by ChromHMM.

A well-established mechanism for eQTLs is that SNPs residing within transcription factor binding sites modulate the expression of nearby target genes. Accordingly, we required PGx-eQTL SNPs to overlap with ER-binding sites identified by ER ChIP-seq performed in breast tissue, where ER is most abundantly expressed. Motif analysis was subsequently conducted on sequences within 200 bp of filtered PGx-eQTL SNPs to determine whether canonical ER-binding motifs were enriched in these regions. Although the ERE(NR)/R3/MCF7-ERα motif was the top-ranked ER-specific motif and was highly enriched (*p* = 1.0 × 10^−36^), the CTCF motif showed the strongest overall enrichment (*p* = 1.0 × 10^−98^), consistent with its established role as a regulator of ER chromatin interactions [21], and *FOXA1* co-binding motifs similarly showed strong enrichment (*p* = 1.0 × 10^−18^), in line with *FOXA1*’s well-documented role as a key determinant of ER-chromatin interactions and endocrine response in breast cancer [22] (Figure 2a).

To shed light on the molecular mechanisms by which these SNPs regulate gene expression, we examined their genomic locations relative to corresponding PGx-eQTL genes. Consistent with our earlier findings in AR- and GR-mediated PGx-eQTL studies [18], most of the SNPs resided in non-coding regions (approximately 99.4%) (Figure 2b). Among these, 885 SNPs (46.1%) were located downstream and 800 (41.7%) upstream of genes, while the remaining 10.50% fell within untranslated regions such as introns (1462; 9.74%), 5′-UTRs (6; 0.31%), and 3′-UTRs (8; 0.42%) (Figure 2b). Only a small number of non-synonymous SNPs were identified within their corresponding PGx-eQTL genes, all classified as benign according to ClinVar. Additionally, SNPs located within promoter regions from ~3000 bp upstream of the transcription start site to the 5′-UTR were identified across a subset of corresponding genes, suggesting potential effects on transcription initiation.

We next asked whether the majority of these SNPs exert their regulatory function by altering the transcriptional activity of nearby enhancer elements and drew on chromatin state annotations from multiple tissues generated by Ernst et al. using the ChromHMM program [23,24], where we retrieved SNP-specific cis-regulatory information from the HaploReg database [25]. Overall, 80% of PGx-SNPs were matched to ChromHMM-annotated data, and among these, 50% overlapped with at least one ER ChIP-seq peak; by examining the positional distribution of these SNPs relative to their associated genes, roughly 80% of matched PGx-eQTLs localized to enhancer regions corresponding to ChromHMM states 6_EnhG and 7_Enh, independent of their distance from the gene body. TSS/promoter states, represented by ChromHMM states 1_TssA, 2_TssAFlnk, and 3_TxFlnk, were more enriched near gene start sites (Figure 2c). Overall, we identified genome-wide PGx-eQTL signals responsive to ER-targeting ligands and specific to ER-mediated transcriptional regulation. Chromatin state annotation further revealed that many of these regulatory variants reside within enhancer or promoter elements, offering mechanistic insight into how non-coding SNPs influence gene expression in an ER-dependent context.

### 2.2. ER-Mediated PGx-eQTL Signals Are Associated with Breast Cancer Prognosis and On-Treatment Estrogen Levels

**Figure 3:**
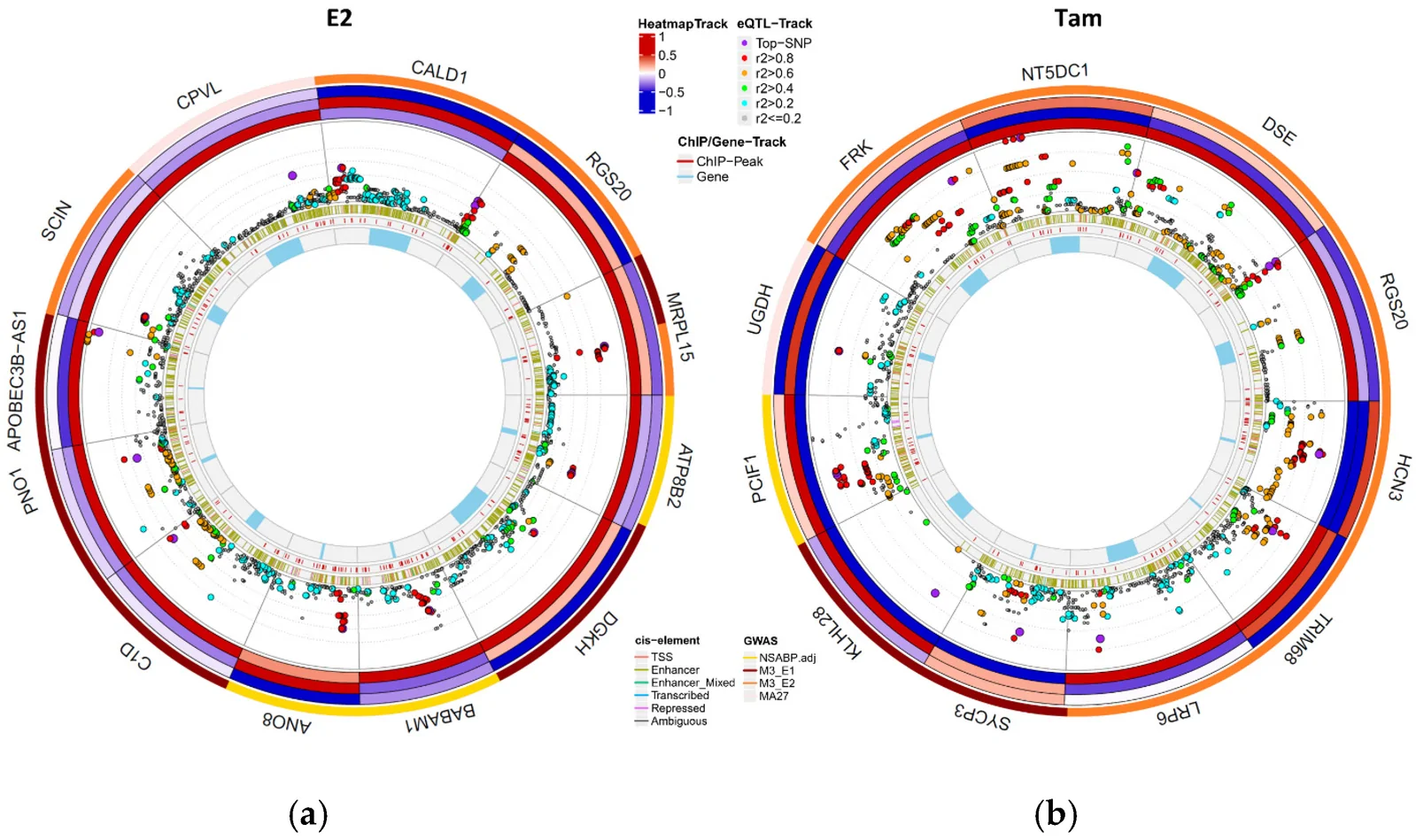
Circos plots in (**a**,**b**) display E2- and TAM-induced PGx-eQTL loci, respectively, implicated in breast cancer GWAS. Each section corresponds to one PGx-eQTL locus. Where multiple PGx-eQTL genes were mapped to the same locus, the top signal was defined as the SNP with the smallest *p*-value located within a ChIP-seq-identified binding site among all signals in that section. The layers, from outer to inner, are as follows: (i) the GWAS study associated with the section’s top PGx-eQTL signal; (ii) three-row heatmaps showing relative gene expression log2-fold changes (drug vs. vehicle) for each of the three genotypes of the top SNP (WT/WT, WT/Alt, Alt/Alt); (iii) *p*-values from the PGx-eQTL analysis, where the x-axis represents genomic coordinates and the y-axis represents -log10(*p*-value), with each log scale marked by a dashed line, and each dot corresponds to a SNP that is a PGx-eQTL for the gene in that section, with the top SNP (the smallest *p*-value SNP within a ChIP-seq-identified binding site) colored purple and all remaining SNPs colored according to their genotypic correlation with the top SNP; (iv) ChromHMM annotation of all SNPs within the locus; (v) ER ChIP-seq peaks; and (vi) the mapped PGx-eQTL gene.

Estrogen receptors are the defining molecular feature of the majority of ER-positive breast cancers, and their role in driving tumor growth and mediating endocrine therapy response is well established. We next tested whether ER-mediated PGx-eQTL SNPs might contribute to breast cancer prognosis and/or endocrine therapy response by assessing their association with breast cancer phenotypes across three previously published genome-wide association studies (GWAS) from our group: (1) the National Surgical Adjuvant Breast and Bowel Project (NSABP), a clinical trial of selective estrogen receptor modulators (SERMs; tamoxifen or raloxifene) for breast cancer risk reduction in high-risk women [3]; (2) the MA27 trial, the largest study evaluating the efficacy of different aromatase inhibitors (AIs) as adjuvant therapy for ER-positive breast cancer after surgical resection [2]; and (3) the Mayo–MD Anderson–Memorial Sloan Kettering (M3) trial of anastrozole as adjuvant therapy in postmenopausal women with resected early-stage breast cancer, which used on-treatment plasma levels of ESR1 and ESR2 as the response phenotype [6]. To improve SNP coverage in LCLs, we included all genotyped SNPs along with SNPs in linkage disequilibrium (LD; r^2^ ≥ 0.80 in the European population) and overlapped these with clinical GWAS results (SNPs with *p* < 0.005). Using these criteria, we identified 12 E2-specific and 11 TAM-specific PGx-eQTL loci (Figure 3a and Figure 3b, respectively), corresponding to a total of SNPs and genes associated with at least one of these GWAS phenotypes. To determine whether the PGx-eQTL signals were specifically enriched in breast cancer GWAS signals rather than arising by chance, genomic region enrichment analysis was performed, where the PGx-eQTL signals were significantly enriched across all GWAS datasets tested, and the most significantly enriched GWAS phenotype was selected given the central role of E2 and TAM in ER signaling.

**Figure 4:**
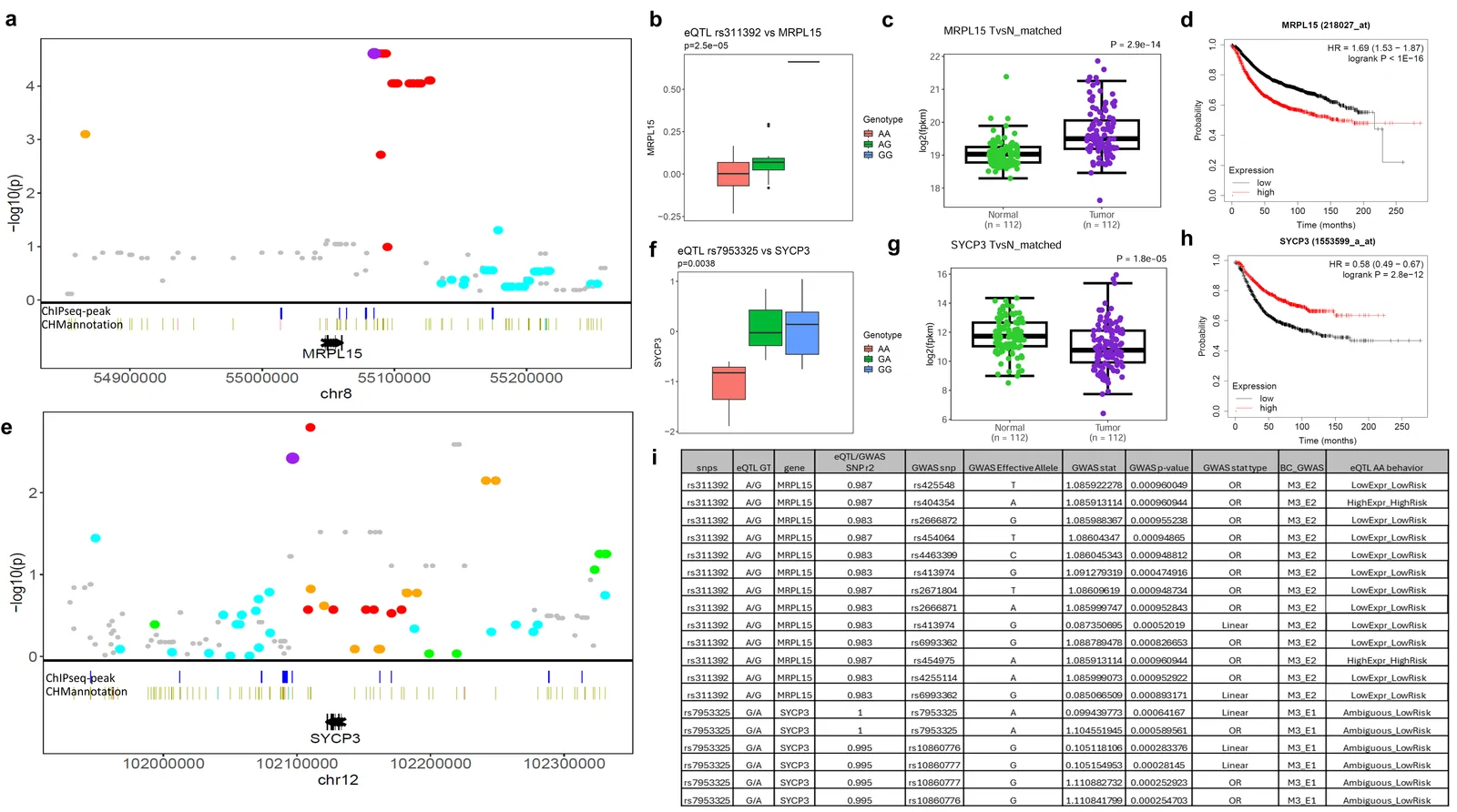
The rs311392-MRPL15 and rs7953325-SYCP3 PGx-eQTL loci were implicated in the GWAS of post-aromatase inhibitor estrogen level and prognosis. (**a**,**e**) How locus zoom plots for the MRPL15 and SYCP3 loci, respectively, with PGx-eQTL results plotted as –log10(*p*-value) against genomic coordinates. The top SNPs (rs311392 for MRPL15 and rs7953325 for SYCP3) are shown in purple, while the other SNPs are color-coded by their genotypic correlation with the top SNP. ER ChIP-seq peaks and ChromHMM annotation of all SNPs at each locus are shown beneath the corresponding SNP plot. (**b**,**f**) E2-induced log-fold change (y-axis) of (**b**) MRPL15 and (**f**) SYCP3 expression respectively stratified by genotype at rs311392 and rs7953325. (**c**,**g**) Compare expression of MRPL15 and SYCP3 between tumor and adjacent tumor-periphery normal tissue in the TCGA breast cancer cohort, with statistical significance assessed by the Mann–Whitney nonparametric test. (**d**,**h**) Relapse-free survival analysis across multiple breast cancer cohorts, grouped by MRPL15 and SYCP3 expression, respectively. (**i**) List of PGx-eQTL SNPs and GWAS signals that are either exactly matched or in LD (r^2^ > 0.8) with the MRPL15 and SYCP3 loci.

Among the identified loci were two genes of particular interest, *MRPL15* and *SYCP3*, which were selected for further downstream functional analysis (Figure 4a,e). Interestingly, *MRPL15* was significantly overexpressed in tumor compared to adjacent normal tissue in the TCGA breast cancer cohort (Figure 4c, *p* = 2.80 × 10^−14^). The A/G genotype of SNP rs311392 was associated with E2-induced increased expression of *MRPL15* (Figure 4b, *p* = 2.5 × 10^−5^), and higher MRPL15 expression was associated with worse relapse-free survival across multiple breast cancer cohorts (Figure 4d, HR = 1.69, *p* < 10^−18^). Although a single nucleotide replacing G with A at locus rs311392, making it A/A, induces downregulation of *MRPL15*, which is associated with better relapse-free survival post E2-treatment (Figure 4b, *p* = 2.5 × 10^−5^), these findings suggest that patients with the rs311392 SNP may experience E2-driven upregulation of *MRPL15*, potentially contributing to poorer outcomes, and may therefore benefit from ER-targeted therapy. In a similar fashion, the SNP rs7953325 was associated with TAM-induced regulation of *SYCP3* expression (Figure 4f, *p* = 0.038) and *SYCP3* was significantly over-expressed in normal breast tissue compared to tumor (Figure 4g, *p* = 1.50 × 10^−5^) and lower *SYCP3* expression was associated with better relapse-free survival (Figure 4h, HR = 0.58, *p* = 2.80 × 10^−12^) Interestingly genotype G/A induces baseline expression of *SYCP3* but a single nucleotide change with replacement of G with A making the A/A genotype reverses the effect by down-regulating TAM-induced *SYCP3* expression (Figure 4f). Taken together, these observations suggest that patients harboring specific genotypes at rs7953325 may be subject to TAM-dependent modulation of *SYCP3* expression, with potential implications for prognosis and endocrine therapy response.

### 2.3. ER-Mediated PGx-eQTL Signals Were Implicated in Breast Cancer Risk and Hormone-Related GWAS Phenotypes

**Figure 5:**
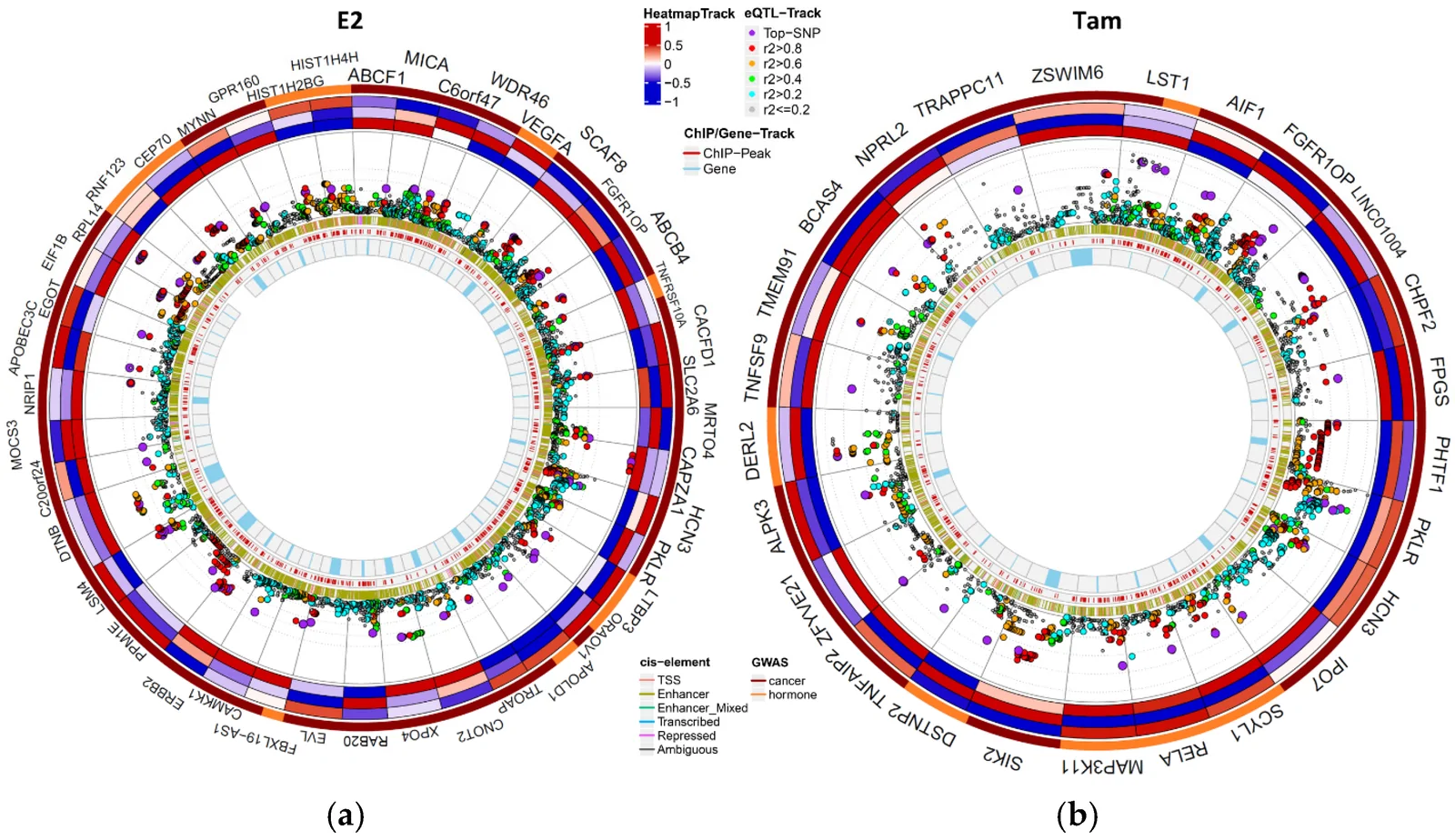
Circos plots for (**a**,**b**) E2- and TAM-induced PGx-eQTL loci implicated in the GWAS catalog for cancer/hormone related phenotypes. Each section represents a PGx-eQTL locus. Where multiple PGx-eQTL genes mapped to the same locus, the top signal was defined as the SNP with the smallest *p*-value located within a ChIP-seq-identified binding site among all signals in that section. The layers, from outer to inner, are as follows: (i) the GWAS study associated with the section’s top PGx-eQTL signal; (ii) three-row heatmaps showing relative gene expression log2-fold changes (drug vs. vehicle) for each of the three genotypes of the top SNP (WT/WT, WT/Alt, Alt/Alt); (iii) *p*-values from the PGx-eQTL analysis, where the x-axis represents genomic coordinates and the y-axis represents -log10(*p*-value), with each log scale marked by a dashed line and each dot corresponds to a SNP that is a PGx-eQTL for the gene in that section, with the top SNP (the smallest *p*-value SNP within a ChIP-seq-identified binding site) colored purple and all remaining SNPs colored according to their genotypic correlation with the top SNP; (iv) ChromHMM annotation of all SNPs within the locus; (v) ER ChIP-seq peaks; and (vi) the mapped PGx-eQTL gene.

**Figure 6:**
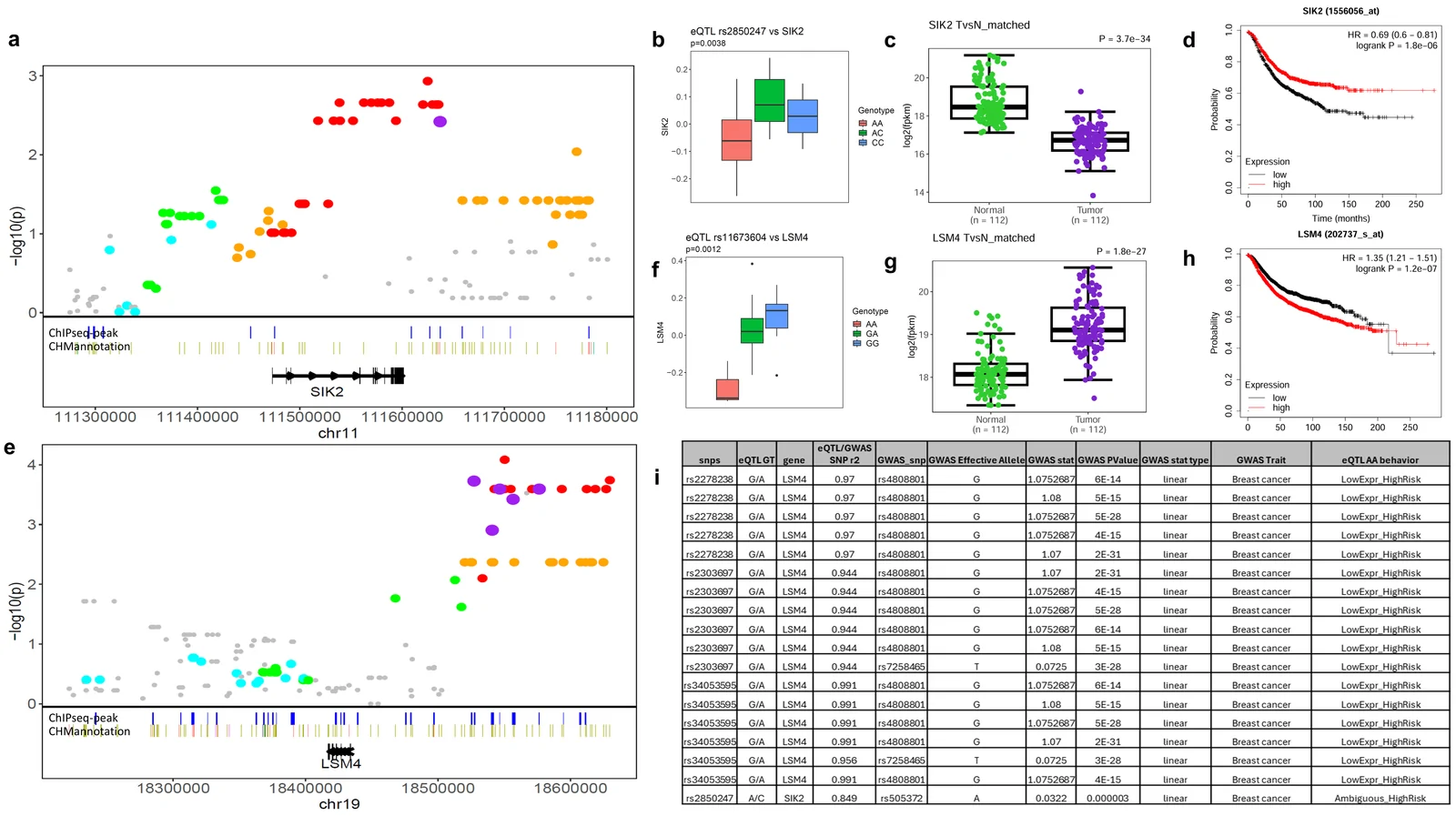
rs2850247-SIK2 and rs11673604-LSM4 PGx-eQTL loci were implicated in GWAS of breast cancer from the GWAS catalog. (**a**,**e**) Locus zoom of (**a**), *SIK2*, and (**e**), *LSM4* loci, respectively. PGx-eQTL is plotted as −log10(*p*-value) for genomic coordinates. The top SNPs (rs2850247 for *SIK2* and rs11673604 for *LSM4*) are depicted in purple, while the remaining SNPs are color-coded by their genotypic correlation with the top SNP. ER ChIP-seq peaks and ChromHMM annotations for all SNPs at each locus are shown beneath the corresponding SNP plot. (**b**,**f**) TAM-induced log-fold change (y-axis) of (**b**)**,** *SIK2*, and (**f**)**,** E2-induced log-fold change (y-axis) of *LSM4* expression by genotype of rs2850247 and rs11673604, respectively. (**c**,**g**) Compare expression of *SIK2* and *LSM4* between tumor and adjacent tumor-periphery normal tissue in the TCGA breast cancer cohort, with statistical significance assessed by the Mann–Whitney nonparametric test. (**d**,**h**) Relapse-free survival analyses across multiple breast cancer cohorts, grouped by SIK2 and LSM4 expression, respectively. (**i**) List of PGx-eQTL SNPs and GWAS signals that either exactly matched or were in LD (r^2^ > 0.8) with *SIK2* and *LSM4* loci.

In the previous section, we investigated the link between endocrine therapy response and ER PGx-eQTLs in breast cancer utilizing data from our three GWAS studies. Now, to identify additional phenotypes for ER-mediated PGx-eQTLs that might be involved in endocrine therapy response, we also performed an overlapping analysis between all the GWAS signals we downloaded from the GWAS database and ER-mediated PGx-eQTLs [26], including SNPs with r^2^ ≥ 0.8 to PGx-eQTL signal SNPs. The ER PGx-eQTL genes mapped broadly across a wide range of traits spanning nearly every aspect of human health, with particular enrichment in tissues and processes known to be regulated by estrogen and ER signaling, such as metabolic regulation, immune function, and reproductive biology, as well as our particular interest, cancer. The eQTL analysis identified 42 and 25 eQTL loci in E2 and TAM, respectively and aligned with ER ChIP-seq peaks and ChromHMM annotation for each locus, and is presented as a circos plot in (Figure 5). Genomic region enrichment analysis was performed for PGx-eQTL loci against GWAS loci from the GWAS catalog, and significant enrichment was identified for two genes of particular interest for breast cancer, *SIK2* (Salt-Inducible Kinase 2) and *LSM4* and presented as regional plots (Figure 6a,e). *SIK2* has been previously reported to maintain breast cancer stemness by phosphorylating *LRP6* and activating Wnt/β-catenin signaling [27]. The SNP rs2850247 was associated with TAM-induced regulation of *SIK2* expression in a genotype-dependent manner (Figure 6b, *p* < 0.05), and *SIK2* was significantly overexpressed in adjacent normal tissue than in tumor in the TCGA breast cancer cohort (*p* = 3.70 × 10^−34^), with higher expression linked to better relapse-free survival (Figure 6c,d, HR = 0.69, *p* = 1.90 × 10^−6^). Interestingly, TAM-induced upregulation of *SIK2* was observed in the A/C genotype at rs2850247 (Figure 6b), which is also associated with improved relapse-free survival across multiple breast cancer cohorts. Although a single nucleotide change replacing C with A, making it A/A, induces TAM-regulated downregulation of *SIK2* (Figure 6b,i), which elevates the risk of breast cancer post-TAM treatment in this disease. Overall, these findings suggest that TAM-mediated modulation of *SIK2* expression in a genotype-dependent manner may have implications for breast cancer risk and treatment response.

Similarly, the SNP rs11673604 was associated with E2-induced regulation of *LSM4* expression (Figure 6f, *p* = 0.012). The G allele of rs11673604 and linked SNPs in rs4808801 (r^2^ = 0.97), were associated with lower *LSM4* expression and lower breast cancer risk (Figure 6i). *LSM4* was significantly overexpressed in tumor compared to normal breast tissue (Figure 6g, *p* = 1.80 × 10^−27^) and higher *LSM4* expression was associated with worse relapse-free survival (Figure 6h, HR = 1.35, *p* = 1.20 × 10^−7^). SNP rs11673604, G/A genotype, was associated with E2-induced upregulation of *LSM4* (Figure 6f), but replacing a single nucleotide G with A through a SNP at the rs11673604 locus, making it A/A, reverses the effect of E2 by downregulating *LSM4* expression (Figure 6f). Taken together, these data suggest that E2-mediated suppression of *LSM4* in a rs11673604 genotype-dependent manner may contribute to breast cancer risk as well as produce a protective effect, potentially through altering *LSM4* expression in tumor tissue.

### 2.4. qRT-PCR Validation of Top ER-Mediated PGx-eQTL Signals in Selected LCL Panel

To validate the robustness of the top PGx-eQTL signals identified from RNA-seq, qRT-PCR experiments were performed for *MRPL15*, *SYCP3*, *SIK2*, and *LSM4* in a selected LCL panel of six ER-expressing LCL cell lines (CA02, CA03, CA05, CA17, CA67 and CA93) to ensure representation of all three genotype categories such as, homozygous variant (2), heterozygous (1), and wildtype (0), for each of the top PGx-eQTL SNPs (rs311392 for *MRPL15*, rs7953325 for *SYCP3*, rs2850247 for *SIK2*, and rs11673604 for *LSM4*). The genotypes of these top PGx-eQTL SNPs across all six cell lines are summarized in Supplementary Table S1, where 0 denotes wildtype (homozygous reference), 1 denotes heterozygous, and 2 denotes homozygous variant genotype. All of the six LCL cell lines were treated with E2, tamoxifen, E2 + tamoxifen and vehicle. Consistent with the PGx-eQTL results, E2-induced upregulation of *MRPL15* was observed across all three cell lines in a genotype-dependent manner, with CA02 being the wildtype and CA05 and CA17 being heterozygous, whereas the effect was reversed in the homozygous CA67 LCL line. For SYCP3, only CA05 showed TAM-induced downregulation of *SYCP3*, which is consistent with its genotype being homozygous at rs7953325. TAM-induced downregulation of *SIK2* in homozygous (A/A) cells is more compared to heterozygous LCL cells, showing functional validation of our initial analysis. Similarly, E2-induced upregulation of *LSM4* was consistently observed in CA02, CA03 and CA17 being heterozygous, whereas the effect was reversed in the homozygous CA93 LCL line. Taken together, these qRT-PCR results in independent cell lines reinforce the reproducibility and genotype-specificity of the ER-mediated PGx-eQTL signals identified in this study.

## 3. Discussion

Individualized medicine depends on understanding why the same treatment produces vastly different outcomes across patients; the genetic context and environmental factors, including endogenous hormone levels and pharmacological treatment, are central determinants of this variability, shaping how transcription factor-mediated gene regulation ultimately influences treatment efficacy. In this manuscript, we have identified genetic factors that may potentially influence the effectiveness of endocrine therapies, including TAM- and E2-mediated transcriptional regulation. Specifically, we showed that E2 upregulated the expression of *MRPL15* and TAM downregulated *SYCP3* in an SNP genotype-dependent manner, and that these genes were associated with post-treatment estrogen levels and relapse-free survival in ER-positive breast cancer patients. Furthermore, TAM-mediated regulation of *SIK2* and E2-mediated regulation of *LSM4* were identified as additional genotype-dependent PGx-eQTL signals with implications for breast cancer risk and prognosis.

*MRPL15* (Mitochondrial Ribosomal Protein L15) is a component of the mitochondrial ribosome and has recently been identified as a novel prognostic biomarker and therapeutic target in ovarian cancer, showing its potential as a biomarker [28]. This mitochondrial marker MRPL15 has also been reported to predict recurrence, metastasis, and TAM resistance in breast cancer patients, suggesting its potential utility as a companion diagnostic for early detection of treatment failure [29]. In our study, E2-induced upregulation of *MRPL15* in a rs311392 genotype-dependent manner, combined with its overexpression in tumor compared to normal breast tissue and its association with worse relapse-free survival, collectively points to a role for *MRPL15* in ER-driven tumor progression and its potential as a prognostic biomarker of this disease. Given the established link between mitochondrial dysfunction and endocrine therapy resistance, E2-mediated induction of *MRPL15* in susceptible genotype carriers may represent a mechanism contributing to estrogen and TAM resistance and disease recurrence. Thus, patients harboring specific genotypes at rs311392 may therefore benefit from closer monitoring. Moreover, considering that resistance in this context appears linked to TAM treatment specifically, an alternative therapeutic strategy, such as aromatase inhibitors or the addition of targeted agents such as CDK4/6 inhibitors, which are established therapeutic options for overcoming endocrine resistance in ER-positive breast cancer, may be considered [30,31]. This warrants further investigation in genotype-stratified clinical studies with a larger patient cohort.

On the other hand, SYCP3 (Synaptonemal Complex Protein 3) is a meiosis-specific protein that has been reported to modulate the level of genome integrity in cancers by impairing mitotic recombination by interfering with *BRCA2* [32,33]. Although *SYCP3* is normally restricted to meiotic germ cells, its aberrant expression in somatic breast tumor tissue is consistent with a cancer-testis antigen-like pattern, in which genes with normally germline-restricted expression become epigenetically reactivated in tumors through a demethylation-dependent process [33]. In our study, TAM-induced regulation of *SYCP3* in a rs7953325 genotype-dependent manner, together with its overexpression in breast tumor tissue and its association with improved relapse-free survival (HR = 0.58, *p* = 2.80 × 10^−12^), suggests that TAM-driven *SYCP3* expression may paradoxically exert a protective role in ER-positive breast cancer. The ability of *SYCP3* to interfere with *BRCA2*-mediated recombination in somatic cells may represent a context-dependent mechanism that limits aberrant DNA repair and tumor progression, though the precise biological basis of this protective association warrants further investigation.

Moreover, by mining the GWAS catalog database, we also identified two additional loci of interest, *SIK2* and *LSM4*, that were implicated in breast cancer risk, where *SIK2* overexpression was associated with better relapse-free survival (HR = 0.69, *p* = 1.80 × 10^−6^). In this study, we observed that TAM-induced expression of *SIK2* occurs in a (rs2850247) genotype-dependent manner, where overexpression was linked to the A/C genotype and improved relapse-free survival, but with the A/A genotype, it reverses the effect by lowering the expression, suggesting a paradoxically protective and enhanced risk association of *SIK2* in breast cancer. While *SIK2* has been linked to cancer stemness through Wnt/β-catenin activation, its higher expression being associated with better outcomes may reflect a context-dependent function in ER-positive breast cancer. TAM-mediated genotype-dependent modulation of *SIK2* expression may therefore represent an important determinant of individual treatment response, warranting further investigation into its precise role in endocrine therapy. Whereas a previous study showed that the other locus of interest, *LSM4*, plays a crucial role in triple-negative breast cancer progression, underscoring its potential as a prognostic biomarker in this disease [34]. We also observed that *LSM4* was significantly overexpressed in tumor compared to normal breast tissue, and higher *LSM4* expression was associated with worse relapse-free survival (HR = 1.35, *p* = 1.20 × 10^−7^), pointing to an oncogenic role in breast cancer. Interestingly, E2-induced *LSM4* overexpression is associated with the G/A genotype and linked to worse relapse-free survival. However, a single SNP at the rs11673604 locus replacing G with A reverses the action of E2 by down-regulating *LSM4* expression. Eventually, this down-regulation of *LSM4* due to E2 treatment improves the overall survival rate of breast cancer. Overall, these findings showed a paradoxical relation between E2 treatment and patient genotypes, where E2-mediated downregulation of *LSM4* in susceptible genotype (A/A) carriers may therefore contribute to best outcomes through reduction of its oncogenic effects in tumor tissue.

Although breast cancer tissue is the most relevant tissue for this study, a well-characterized panel of breast cancer cells suitable for inheritable SNP-dependent pharmacogenomic studies is not currently available. Commercially available breast cancer cell lines, despite their widespread use in drug response studies, are not ideal for this purpose due to their genomic alterations and inherent heterogeneity, which confound the study of SNP-dependent responses to natural ligands. The LCL panel we employed for this study has been extensively characterized by our group and others. These LCLs represent a broad spectrum of human genomic diversity and have been widely used in multiple pharmacogenomic studies, particularly for the studies of context-dependent drug response [35,36,37]. We acknowledge, however, that LCLs are B-lymphocytes, and ER signaling and the associated chromatin-binding landscape are highly tissue-specific. Therefore, ER-mediated transcriptional regulation observed in this LCL model system may not fully recapitulate the regulatory networks operative in breast epithelial tumor cells, even though candidate signals are filtered against breast tissue ER ChIP-seq data. Moreover, we chose to cross-validate our signals with biologically informative datasets by filtering PGx-eQTL signals with ChIP-seq-validated ER binding sites in breast tissue and examining clinical relevance using breast cancer GWAS data rather than relying solely on statistical thresholds. This strategy, previously employed successfully in our PGx-eQTL studies of the glucocorticoid and androgen receptors [18,19], allowed us to prioritize functionally relevant signals. Most GWAS signals reside in non-coding regulatory regions, and their contribution to gene function, disease risk, and prognosis often remains unclear. Our approach bridges this gap by identifying the biological and clinical relevance of PGx-eQTL signals validated against breast cancer GWAS data, suggesting that these SNPs are likely to influence disease or treatment response through their effects on gene expression in the presence of specific hormones, a phenomenon that warrants further investigation. We also note that LCL-specific ER ChIP-seq data were not generated or incorporated in this study; such validation was beyond the scope of the current work and represents an additional limitation to be addressed in future studies. Nonetheless, given the modest sample size and the absence of formal multiple testing correction, this study should be regarded as hypothesis-generating rather than confirmatory, and the PGx-eQTL signals identified here will require validation in larger, independent clinical cohorts before their clinical utility can be firmly established. Additionally, our qRT-PCR validation compared expression across a limited number of genetically distinct LCLs. Although *GAPDH* normalization controlled for technical variability, it does not account for background genetic differences between donors that could also influence expression independent of the SNP studied. Isogenic models or larger genotype-stratified panels would more robustly isolate this SNP-specific effect and represent an important direction for future validation.

## 4. Methods

### 4.1. Experimental Design for ER Modulator-Induced PGx eQTLs

To study the gene-associated effect of ER-mediated PGx-eQTLs, we used human lymphoblastoid cell lines (LCLs). The LCLs are well-known models for genotype-associated pharmacogenomic studies and are well characterized by genotyping [19,35,38]. However, there are variable expressions of ER in different LCLs, so we selected 30 LCLs that have significant expression of ER to study the effect of hormone treatment. After exposing the LCLs to E2, TAM, or both (E2 + TAM), RNA sequencing of treated LCLs was performed. Furthermore, a gene expression analysis to identify PGx-eQTLs was performed following our previously published protocol [19]. In brief, to ensure the PGx-eQTL signals were indeed derived from drug treatment, we selected signals that were reversed from a single treatment to dual treatment. Moreover, we ensured that all signals were located within genomic regions that contain at least one ER-binding site that has been identified through ER ChIP-seq analysis on breast tissues. Finally, the clinical relevance of these ER-mediated PGx-eQTLs was confirmed by overlapping with SNPs published in previous GWAS studies [2,3,6,18,39].

### 4.2. Cell Culture and Drug Conditions

LCLs obtained from the Coriell Institute were maintained in 15%FBS and 1% penicillin-streptomycin-supplemented RPMI-1640 medium in a 5% CO_2_ atmosphere. To treat the LCLs with E2, TAM, and E2 + TAM, cells were cultured for 24 h in 5% charcoal-stripped FBS-supplemented phenol red-free medium followed by 10 nM E2 (Medchem Express, Monmouth Junction, NJ, USA, Cat# HY-B0141), 250 nM TAM and E2 + TAM with vehicle. Hormones and drugs were added to the culture medium and incubated for 8 h before harvesting by centrifugation for RNA extraction.

### 4.3. RNA-Sequencing Experiments

Total RNA was extracted using the RNeasy RNA extraction kit (Qiagen, Germantown, MD, USA, Cat# 74104) following the manufacturer’s instructions, and RNA integrity was assessed using an Agilent Bioanalyzer. RNA-seq libraries were then prepared using the TruSeq2 RNA Library Preparation Kit v2 (Illumina, San Diego, CA, USA), and paired-end sequencing (100 bp) was performed on an Illumina HiSeq 4000 platform at an approximate depth of 25 million paired-end reads per sample. Raw RNA-sequencing reads were aligned with the human reference genome GRCh37 (hg19) using STAR [40], with gene-level counts subsequently generated using HTSeq [26]. For downstream PGx-eQTL analysis, genes with raw counts lower than 32 under any treatment condition were removed. Overall, 8571 genes were excluded through this filtering step, and the remaining 13,741 genes were retained for PGx-eQTL analysis. Moreover, gene expression counts were normalized to library size using the EdgeR package (version 4.2) [41], and expression values were then converted to log2 fold changes relative to the vehicle-treated condition for each cell line.

### 4.4. LCL Genotype Data Quality Control

LCLs derived from unrelated individuals were genotyped using two Illumina platforms, such as the HumanHap550K and HumanExon510S-Duo BeadChips, and analyzed using publicly available data generated and deposited from our laboratory previously under GEO accession GSE23120 [35]. To confirm sample integrity, identity-by-descent analysis was performed using plink-genome, which ruled out any cryptic relatedness or population stratification across individuals. Furthermore, genotypes were then subjected to quality control filtering in plink (https://www.cog-genomics.org/plink/1.9/) [42]. For ER-expressing cells specifically, the following thresholds were applied: a minor allele frequency (MAF) of at least 0.2, a maximum missingness rate of 5%, and a Hardy–Weinberg equilibrium *p*-value exceeding 0.001.

### 4.5. Pharmacogenomic eQTL Analysis

PGx-eQTL gene expression was analyzed using the R package MatrixEQTL with an ANOVA model, and results were reported as fold change in the drug treatment group relative to the vehicle control group [43]. Given the limited sample size (*n* = 30 LCLs) and the corresponding constraints on statistical power, a nominal significance threshold of *p* ≤ 0.005 was applied without formal correction for multiple testing. The analysis focused on cis-eQTL SNPs located within 200 kbp upstream or downstream of the corresponding gene in LCLs. Trans-eQTLs, as well as variants on the sex chromosomes and mitochondrial genome, were excluded from all analyses. To refine the analysis, signals classified as ambiguous or treatment-independent were identified in the E2-TAM combination treatment condition, due to the fact that E2 and TAM are known to act as antagonists in ER signaling. Signals that persisted under combination treatment were excluded to ensure the specificity of results. Subsequently, ER ChIP-seq datasets were used to map ER binding sites and to pinpoint PGx-eQTL SNPs with high confidence.

### 4.6. ER ChIPseq Data

Remap2022 (https://remap2022.univ-amu.fr/) accesse on Octiober 2023 [44] was used to download human ER ChIP-seq peak data. Experiments incorporated both normal and malignant breast and prostate tissues, as well as corresponding cell line models. The data were mapped to the hg38 reference genome and were converted to hg19 using LiftOver tools from the UCSC genome browser website (https://genome.ucsc.edu/cgi-bin/hgLiftOver, accessed on 17 April 2023). We applied the GenomicRanges package in RStudio to systematically integrate ChIP-seq profiles with PGx-eQTL SNP datasets [45].

### 4.7. ChromHMM Annotation of SNPs

ChromHMM annotation data were obtained from HaploReg4.2 (version 4.2) (https://pubs.broadinstitute.org/mammals/haploreg/haploreg.php) [25], which contains multi-tissue ChromHMM 25-states and 15-states annotation of SNPs [23,24]. The 15 chromatin states defined by ChromHMM were grouped into four functional categories: TSS (1_TssA and 2_TssAFlnk, which represent transcription start sites), Transcription (3_TxFlnk, 4_Tx, and 5_TxWk, representing active transcription), Enhancer (6_EnhG and 7_Enh, indicating enhancer activity), and Repressed (including all remaining states, heterochromatin, bivalent or poised TSS/enhancers, and other inactive chromatin). Given the limited number of SNPs carrying breast tissue-specific annotations, chromatin states for each SNP were aggregated across all available tissues, and the most frequently occurring annotation category was assigned as the representative state. This grouping strategy was necessary to ensure broader and more reliable chromatin state coverage. However, when two active states appeared, the SNP was designated as Enhancer_Mixed; cases in which an active and a repressed state were represented equally were classified as Ambiguous.

### 4.8. Homer Motif Analysis

To define motif regions, the genomic coordinates of significant SNPs identified from the PGx-eQTL analysis were extended by 200 bp in both the upstream and downstream directions. For the ChIP-seq filtered motif analysis, an additional layer of specificity was applied; only SNPs that directly overlapped with ER ChIP-seq peaks were retained, and these were used to generate the final motif regions. Furthermore, motif enrichment analysis was carried out using the findMotifsGenome.pl command within the HOMER software suite [46].

### 4.9. SNPs Overlapping with GWAS Signals

Genome-wide association studies relevant to this work have been previously reported, including: breast cancer prevention by SERMs in the NSABP trial (592 cases, 1171 controls, all Caucasian) [3]; MA.27 adjuvant trial comparing AI exemestane and anastrozole (252 cases, 4406 controls; 95.7% Caucasian, 3.0% African, 1.3% Asian) [2]; and circulating estradiol (E2) and estrone (E1) levels before and during anastrozole treatment in the M3 trial (44 cases, 278 controls, all Caucasian) [6]. From these studies, SNPs with *p*-values ≤ 0.005 were compared with PGx-eQTL SNPs. Supplementary GWAS data were retrieved from the GWAS Catalog (https://www.ebi.ac.uk/gwas/, accessed on 19 December 2023) [39]. Given the relatively limited SNP coverage inherent to the LCL genotyping array, linkage disequilibrium (LD) block analysis was conducted to compensate for this constraint. This was achieved through API queries to the LDlink database (https://ldlink.nih.gov/, accessed on 11 March 2024) using the LDlinkR package in R [47], and SNPs exhibiting an r^2^ value of 0.8 or greater between PGx-eQTL and GWAS variants were considered to occupy the same LD block and were therefore regarded as likely contributors to the same underlying phenotype. Finally, Circos plots were generated using the circlize R package [48], and loci enrichment analysis was performed using the LOLA package in R.

### 4.10. Survival Analysis

KMplotter (https://kmplot.com/analysis/, accessed on 19 April 2024) was used to perform survival analysis [49]. A multi-cohort breast cancer dataset was utilized, with relapse-free survival as the outcome, and gene expression groups were stratified using the software’s automatically determined cutoff.

### 4.11. qRT-PCR Validation Experiments

Six additional ER-expressed LCL cell lines (CA02, CA03, CA05, CA17, CA67, CA93) were cultured and treated with E2, TAM, E2 + TAM and vehicle untreated control. Total RNA was extracted and quantified using Nanodrop as described above. Further, a one-step qRT-PCR was performed using Power SYBR Green (Thermo Fisher) using respective primer pair mix MRPL15 Human qPCR Primer Pair (NM_014175), SCP3 (SYCP3) Human qPCR Primer Pair (NM_153694), Snf1lk2 (SIK2) Human qPCR Primer Pair (NM_015191) and LSM4 Human qPCR Primer Pair (NM_012321) from Origene. Relative gene expression levels were calculated using the ΔΔCt method, with GAPDH used as the internal control. Each experiment was performed in three technical replicates, and expression of the genes had to be detectable by qRT-PCR (ΔCt < 30).

## Data Availability

All the processed data for this manuscript are available at https://doi.org/10.5281/zenodo.22583586.

## Supplementary Materials

The following supporting information can be downloaded at: https://www.mdpi.com/article/10.3390/ijms27188217/s1.
