# Supplementary material for: Study of Estrogen Receptor-Mediated PGx-eQTLs Identifies Genetic Determinants of Breast Cancer Endocrine Therapy Response
Source: Int J Mol Sci. 2026 Sep 15;27(18):8217. doi: 10.3390/ijms27188217 (PMC13607051; doi:10.3390/ijms27188217)
Supplement: Supplementary file 1 [file ijms-27-08217-s001.zip › ijms-4483080-supplementary.pdf]

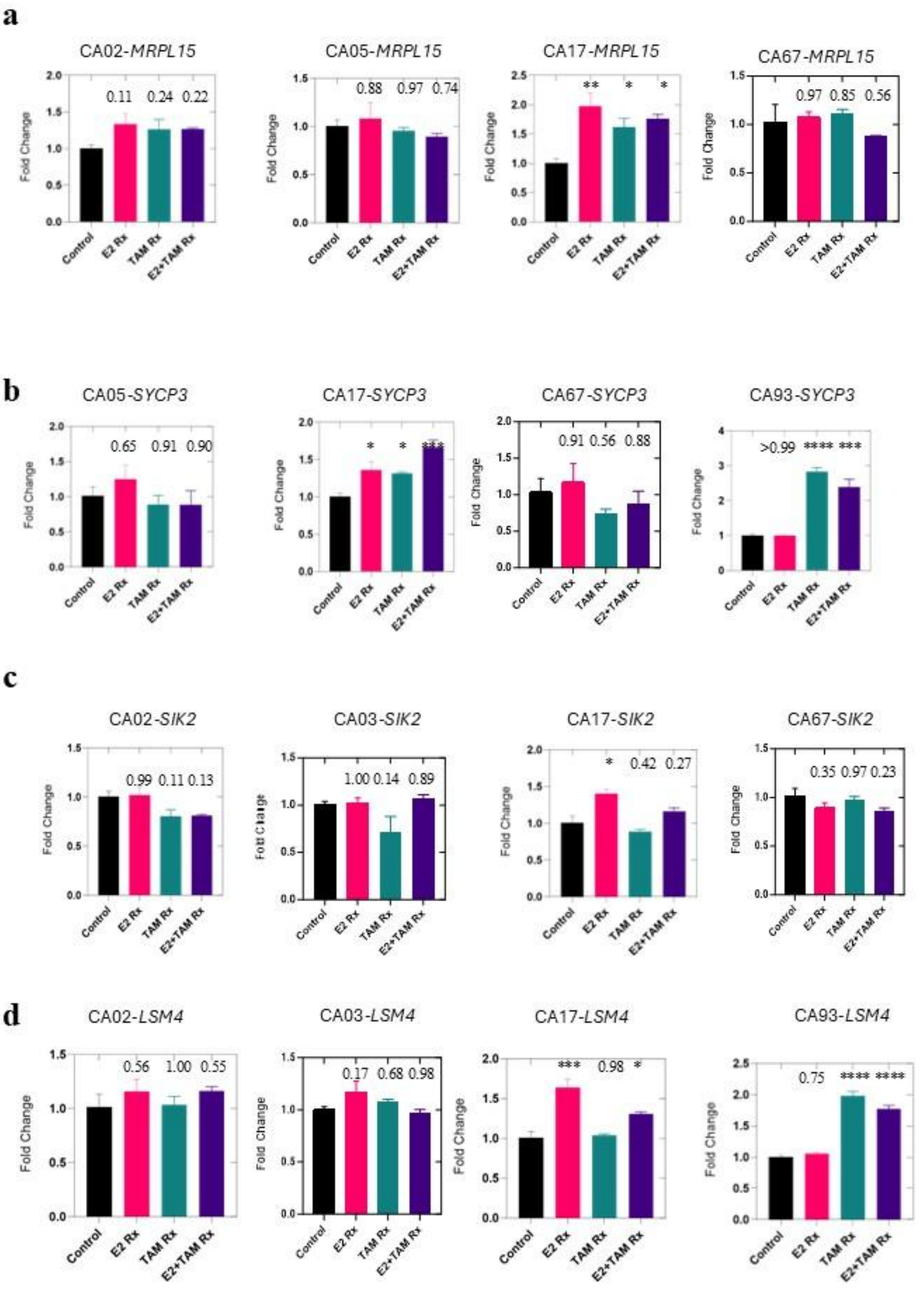
a
CA02-MRPL15
0.11 0.24 0.22
CA05-MRPL15
0.88 0.97 0.74
CA17-MRPL15
** * *
CA67-MRPL15
0.97 0.85 0.56
b
CA05-SYCP3
0.65 0.91 0.90
CA17-SYCP3
* * ***
CA67-SYCP3
0.91 0.56 0.88
CA93-SYCP3
>0.99 **** ***
c
CA02-SIK2
0.99 0.11 0.13
CA03-SIK2
1.00 0.14 0.89
CA17-SIK2
* 0.42 0.27
CA67-SIK2
0.35 0.97 0.23
d
CA02-LSM4
0.56 1.00 0.55
CA03-LSM4
0.17 0.68 0.98
CA17-LSM4
*** 0.98 *
CA93-LSM4
0.75 **** ****
Fold Change
Control
E2 Rx
TAM Rx
E2+TAM Rx

	MRPL15	SYCP3	SIK2	LSM4
**Cell**	**rs311392**	**rs7953325**	**rs2850247**	**rs11673604**
CA02	0	0	2	1
CA03	0	1	1	1
CA05	1	2	0	0
CA17	1	1	2	1
CA67	2	1	1	0
CA93	0	0	0	2
